# Personalised Medicine and the Potential Role of Electrospinning for Targeted Immunotherapeutics in Head and Neck Cancer

**DOI:** 10.3390/nano14010006

**Published:** 2023-12-19

**Authors:** Connor H. O’Meara, Thanh Vinh Nguyen, Zuhayr Jafri, Michael Boyer, David C. Shonka, Levon M. Khachigian

**Affiliations:** 1 Department of Otorhinolaryngology, Head & Neck Surgery, The Canberra Hospital, Garran, ACT 2605, Australia; 2 ANU School of Medicine, Australian National University, Canberra, ACT 0200, Australia; 3 School of Chemistry, University of New South Wales, Sydney, NSW 2052, Australia; t.v.nguyen@unsw.edu.au; 4 Vascular Biology and Translational Research, Department of Pathology, School of Biomedical Sciences, Faculty of Medicine and Health, University of New South Wales, Sydney, NSW 2052, Australia; z.jafri@student.unsw.edu.au (Z.J.);; 5 Chris O’Brien Lifehouse, Camperdown, NSW 2050, Australia; michael.boyer@lh.org.au; 6 Department of Otolaryngology, Head & Neck Surgery, University of Virginia School of Medicine, Charlottesville, VA 22903, USA

**Keywords:** immunotherapy: head & neck cancer, small molecule immunotherapies, neutrophil extracellular trap immunotherapies, nanofiber scaffolds, immunotherapeutic nanofiber scaffolds

## Abstract

Advanced head and neck cancer (HNC) is functionally and aesthetically destructive, and despite significant advances in therapy, overall survival is poor, financial toxicity is high, and treatment commonly exacerbates tissue damage. Although response and durability concerns remain, antibody-based immunotherapies have heralded a paradigm shift in systemic treatment. To overcome limitations associated with antibody-based immunotherapies, exploration into de novo and repurposed small molecule immunotherapies is expanding at a rapid rate. Small molecule immunotherapies also have the capacity for chelation to biodegradable, bioadherent, electrospun scaffolds. This article focuses on the novel concept of targeted, sustained release immunotherapies and their potential to improve outcomes in poorly accessible and risk for positive margin HNC cases.

## 1. Head and Neck Cancers

Head and neck cancer (HNC) is the sixth most common group of malignancies worldwide, with 890,000 new cases and 450,000 deaths in 2018; its incidence continues to rise, with a predicted 30% increase in cases of head and neck squamous cell carcinoma (HNSCC) by 2030 [1,2,3]. Approximately 65% of cases are present in an advanced state [4], commonly requiring functionally and aesthetically deforming procedures that impact the quality of life. Recent research has demonstrated that the incidence of stage IV HNC is increasing even in developed countries [5]. Despite significant therapeutic advances, the average five-year overall survival (OS) has improved little over the past 30 years and remains at approximately 66% [6]. Financially, HNC is also one of the most expensive cancers to manage [7,8]. This places an enormous burden on patients and health systems alike. New and effective therapies beyond surgery alone are therefore keenly sought.

## 2. Nanofibers

Nanofibers are a unique one-dimensional nanomaterial with a variety of physiochemical properties and a cross-sectional diameter that can range from one to hundreds of nanometres [9,10]. These can be produced from many materials and are characterised by small pore diameter, high porosity, and a high surface area-to-volume ratio [11,12,13]. This suggests versatile use in a range of biomedical applications. Nanofiber production can be achieved using the following techniques: template synthesis, self-assembly, phase separation, and electrospinning [14,15,16,17,18,19]. These are addressed in turn below.

## 3. Template Synthesis

Nanoporous membranes are used in template synthesis to create nanofibers that are either solid or hollow. For example, Deeney and colleagues created luminous carbon nanofibers using microwave pyrolysis of polyethyleneimine and citric acid with a template [20]. A dense vertical network of nanofibers averaging a diameter of 200 nm was generated. Similarly, Li and colleagues utilised a soft-template technique, producing a microporous device with a nanofiber three-dimensional structure [21]. These nanofibers were created from Li_2_FeSiO_4_/C with diameters ranging from 20 to 30 nm. A key benefit of this technology is the ability to produce nanofibers from various base materials, namely metals, polymers, and carbons [20,21]. However, a significant limitation of this method is the inability to generate nanofibers continuously and sequentially [22].

## 4. Self-Assembly

Nanofibers can be created by self-assembly via self-organisation of pre-existing components, namely proteins or peptides [23,24]. Unfortunately, self-assembly is comparatively ineffective for producing continuous polymeric nanofibers [25,26]. In a chemistry first, Chen and colleagues utilised chalcogen-bonding interactions to create nanofibers with quasi-calix-4 chalcogenadiazole (C4Ch) serving as a macrolide donor with a tailed pyridine N-oxide surfactant serving as a molecule acceptor. TeO or SeO chalcogen-bonding interactions and the self-assembly process produced the nanofibers. The creation of a one-dimensional fibre network with a uniform radial diameter of 6.5 nm was confirmed by TEM imaging [27].

## 5. Phase Separation

Both self-assembly and phase separation are comparatively slow techniques for producing continuous nanofibers [28,29]. Phase separation requires polymer dissolution, gelation, extraction with a different solvent, freezing, and, ultimately, drying [30]. This process produces a foam with nanoscale pores. For example, Zhao and colleagues created a nanofiber made of chitosan acetate using solid–liquid phased separation. Of importance, temperature, acetic acid, and chitosan concentrations are all capable of altering nanofiber structure. The study demonstrated the production of nanofibers of 50 to 500 nm size in the presence of 0.05% (*w*/*v*) chitosan and 0.024% (*v*/*v*) acetic acid in liquid nitrogen [31].

## 6. Electrospinning

**Figure 1:**
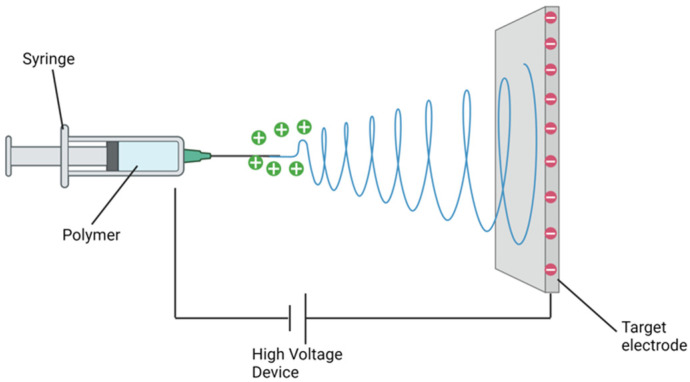
Schematic representation of electrospinning apparatus.

Electrospinning is an efficient, inexpensive, and technically straightforward means of producing nanofibers. Fibres are produced essentially by applying an electric field across a polymer mixture [32,33,34]. The following outlines the minimal apparatus required to develop a simple electrospinning facility: a viscous polymer solution, an electrode (solid, hollow or tubular) that is kept in contact with the polymer solution, the electrode connected to a high-voltage DC generator, and to collect the nanofibers, a grounded or oppositely charged surface [19]. A syringe pump affords a consistent, steady flow of polymer [35]. The intent of this electrostatic technique is to induce the ejection of a liquid jet towards a grounded surface known as a collector by applying a high voltage field strength that starts as low as 1 KV cm^−1^ to the polymer solution droplet surface being held at the end of a spinneret or syringe needle, known as the Taylor cone effect [36,37] (Figure 1).

The Taylor cone effect identified that a single jet would initially split into several filaments when the critical voltage (CV) is reached via radial repulsion [13]. This splay of filaments evaporates the solvent, hardening nanofibers and landing upon the recipient plate. Importantly, the critical voltage differs between base polymers [38]. Ultimately, the configuration or shape of the produced fibres depends upon several operational factors, namely electric field intensity, jet distance, and flux, inclusive of polymer solution variables: viscosity, concentration, volatility, and dielectric constant.

Many polymers can be difficult to electrospin; however, optimising experimental strategy and set-up can mitigate many of the associated issues [19,39]. ‘Up-scaling’ or producing continuous nanofibers on an industrial scale can also be achieved by utilising multi-jet electrospinning devices that have been reported to process at least 6.5 kg/h of polymer to create uniform nanofibers, improving the potential for commercially viable products from laboratory experiments [40,41]. Unfortunately, ‘up-scaling’ is not achievable with all ‘polymer to nanofiber’ configurations.

Currently, there are several commercially available products generated from electrospinning. These include the Zeus Bioweb, a polytetrafluoroethylene (PTFE)-based composite [42]. The Bioweb has a large surface area and a pore consistency of 1–4 nm. It has a variety of described applications, including endovascular stent coatings, scaffolds for drug loading, and implantable body structures [42]. Similarly, SpinCare, produced by Nanomedic, is another commercially available electrospun scaffold. SpinCare is a portable device utilised to create nanofibers for wound healing. The produced nanofiber dressing has several useful characteristics; a semi-permeable layer allowing for moisture regulation, can be tailored to a wound and reduces microbial infection [43].

## 7. Pharmacology Loading onto Electrospun Nanofibers

**Figure 2:**
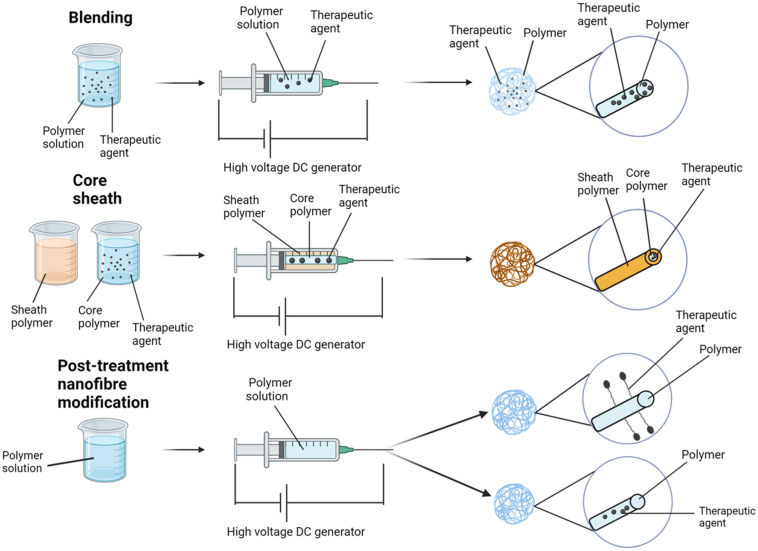
Common techniques for loading therapeutic agents onto electrospun nanofibers.

The potential for loading drugs onto nanofiber scaffolds represents a paradigm shift in the targeted delivery of therapies to pathology, especially malignancy. To date, excipient loading onto nanofiber scaffolds has demonstrated potential in wound care [13,17,18], diabetes [44,45,46], and cancer [47,48,49,50]. There are four typical techniques for loading therapies onto electrospun nanofibers: blending, core–sheath, encapsulation, and chemical immobilisation (Figure 2).

## 8. Blending

Blending is the most common approach and involves initially dissolving or dispersing the therapeutic agent within the polymer solution [51]. Consequently, drug release is influenced by molecule distribution within the fibres, as well as the fibres’ morphological characteristics [52]. In this technique, consideration must be given to the interaction between the drug and the loadable polymer, as this can influence encapsulation efficacy, drug dispersal and sustained release profile. It is also imperative to consider the effect of solvent exposure on the therapeutic agent/s. Given that blending preserves nanoparticle integrity in the setting of being quick and easy, there is a preference for its use in products incorporating metal-based therapies [53]. For instance, silver nanoparticles are an effective anti-septic and antibacterial therapy [54]. Polyacrylonitrile (PAN) electrospun nanofibers loaded with silver nanoparticles were developed by Rujitanaroj and colleagues, who demonstrated that PAN scaffolds lacking silver nanoparticles failed to impair *S. aureus* or *E. coli* growth. Comparatively, when compared to vancomycin and gentamycin (for *S. aureus* and *E. coli*), silver nanoparticle PAN scaffolds demonstrated significant and comparable zones of bacterial inhibition (*p* < 0.05) [55].

## 9. Core–Sheath

Co-axial or emulsion electrospinning produces core–sheath nanofibers [56,57]. Co-axial electrospinning is a dual-stream technique for creating multipolymer fibres; the internal stream serves as the ‘core’, and the outer serves as the ‘sheath’. Loading the ‘core’ affords protection by the ‘sheath’, mitigating potential loss of the loaded therapeutic agent/s, especially if they are at risk of unwanted degradation [58]. In addition, this technique attenuates therapeutic contact with the sheath polymer blend, which is commonly produced with solvents, protecting the loaded nanofiber scaffold core. Investigators have recognised the potential benefit of this method for gene delivery due to such properties [58,59]. It is therefore imperative to inhibit loaded genetic material exposure to organic solvents and excessive voltages. In electrospinning, a non-woven fibre matrix, Luu and colleagues encapsulated condensed DNA into a poly(lactic co-glycolic acid) (PLGA) core shielded by a PLA sheath. DNA was protected from degradation, remained viable and demonstrated sustained release over 20 days. Approximately 69–80% of the loaded DNA was ultimately released from the nanofiber scaffold [60].

Emulsion electrospinning, in contrast to co-axial, utilises a single nozzle to create nanofibers from two immiscible liquids with core–sheath morphologies [61]. This technique relies upon chemical separation, accomplished by the addition of an emulsion to the initial polymer solution and utilising surfactant to keep the phases apart. Emulsion is commonly utilised for the loading of delicate biological molecules (i.e., enzymes or growth factors) [59]. The emulsion can accomplish the protection of biomolecules or hydrophilic drugs from solvents that commonly reside in the sheath part of the fibres by dissolving them in the water phase of a water-in-oil emulsion.

Utilising DNA-based nanoparticles and chitosan loaded on nanofiber or encapsulated within HA/PLGA solution prior to electrospinning, Nie and colleagues developed electrospun nanofibers made of PLGA/HA containing naked pDNA (encoding BMP-2) [62]. Using human mesenchymal stem cells (hMSC), these researchers found that hydrophilic HA accelerated pDNA release, increasing hMSC adhesion. When scaffold transfection efficacy was studied using hMSCs, it was noted that loading of nanoparticles post-electrospinning was the only viable technique for boosting BMP-2 transgene expression. Interestingly, the scaffold appeared to exhibit a decline in cell viability over time. Intense transfection of the nanoparticles produced from chitosan and pDNA was identified as the cause of this issue. Although such findings using emulsion are encouraging, it is important to note, in contrast to co-axial electrospinning, that there is potential for emulsion to harm macromolecules (pDNA) secondary to shearing force and tension between the two phases.

## 10. Attachment of Potential Therapeutics

Another well-described technique is the modification of the electrospun nanofiber surface to improve the integration and attachment of therapeutic agents. By utilising this technique, devices can be developed that modify release dynamics and specifically decrease burst release for loaded therapeutic scaffolds [63]. Importantly, surface conjugation and gradual release help loaded drugs (especially enzymes and gene therapy) retain their biological function against strong solvents and voltage exposure.

Efficacious targeted delivery of therapeutic agents has been proposed as a significant benefit of combining gene therapy with biomaterials. In line with the above, electrospun biomaterials show promise for gene delivery in regenerative medicine. For example, local targeting of upregulated genes associated with wound chronicity can be downregulated by delivery of gene therapy utilising siRNA. The effective preservation of siRNA-MMP-2 from degradation was successfully achieved via a gene delivery system: linear polyethyleneimine (LPEI). MMP-2 dysregulation is associated with chronic wounds. In these experiments, LPEI-siRNA complexes were immobilised upon PCL and PEG nanofibers and delivered which led to MMP-2 inhibition, promoting chronic wound healing. Comparing untreated wounds and those treated with nanofiber scaffold alone or nanofiber scaffolds loaded with LPEI-siRNA, researchers inhibited MMP-2 expression (*p* < 0.01) and improved wound healing [64].

## 11. Post-Treatment

There is potential for therapeutics to be synthesised within nanofiber scaffolds after the electrospinning process is completed. Commonly metallic, this can be achieved by electrospinning metallic precursor material within the preferred polymer solution, with subsequent procedures facilitating nanoparticle creation. A benefit of this approach is continuous or sustained release of therapeutic agents, either by diffusion or degradation and release from the nanofiber scaffold.

Gas–solid interaction is a technique of post-treatment loading where nanofiber exposure (to a specific gas environment) catalyses the reaction. First described by Wang and colleagues in the setting of electrospun polyvinylpyrrolidone (PVP), lead ion nanofibers of average 5 nm size [65] were produced within the fibres subsequent to exposure to H_2_S gas at room temperature. Similarly, Yang and colleagues investigated PAN nanofibers electrospun from AgNO_3_ and exposed them to an environment of hydrochloric acid (HCl), facilitating the general distribution of AgCl nanoparticles throughout the scaffold [66].

A further technique is plasma treatment, which modifies the chemistry of a hybrid matrix and provides a platform for nanoparticle synthesis [67]. To date, nanoparticle development by plasma treatment is thought to be a straightforward and effective process that is also ecologically friendly. Utilising PAN and AgNO_3_ precursor, and PAN HAuC_14_ precursor solutions, Bei and colleagues produced electrospun nanofibers at 50 W, 100 mTorr, and 10 cm^3^/min; both varieties of nanofiber were treated using an argo plasma laser. Both approaches were deemed to generate appropriately sized nanoparticles for systemic delivery [66].

## 12. Electrospun Nanofibers in Cancer Therapy

Population statistics predict progressive increases in cancer incidence over the coming decade, 72% since 2008 [68]. Therapy for HNC has changed little in the last 50 years and is associated with de-forming/functioning surgical procedures and radiotherapy, with systemic chemotherapy as required. However, systemic therapy can have significant off-target side effects [69]. Novel targeted therapeutic strategies are a necessity to mitigate these issues.

There are several instances in head and neck oncology that would benefit from targeted delivery of pharmacologically loaded nanofiber scaffolds. In the setting of surgical resection, proximity to important functional anatomy (i.e., eye) may result in a surgical decision to reduce the resection margin, increasing the likelihood of a close or involved margin and risk of ongoing disease, recurrence or necessity for adjuvant therapy which may injure the structure of concern (i.e., radiation-induced optic neuropathy) [70]. Loaded nanofibers could be delivered intra-operatively to the site of potential or positive margin promoting residual cancer cell death, mitigating recurrence without the necessity for radiotherapy or systemic therapies [17,71].

Many polymers have demonstrated potential as nanofibers in the setting of cancer prevention and treatment. For example, utilising electrospun nanofibers as a cell capture device may enable earlier and more accurate oncology diagnosis and treatment. Zhang and colleagues developed TiO_2_ nanofibers expressing EpCAM and coated upon a silicon substrate to investigate colorectal cancer diagnosis, where utilised colorectal cell lines (BGC823 and HCT116) are both noted to express EpCAM [72]. To determine efficacy, peripheral blood was collected from patients suffering from colorectal and gastric cancers. Systemically circulating cancer cells were detected as captured, or otherwise, by three colour immunohistochemical techniques. Experiments concluded that circulating cancer cells ranged from 0 to 19/0.5 mL blood in colorectal (2/3) and gastric cancer (7/7) patients. Consequently, confirming an oncological diagnosis with an inexpensive and non-invasive test would be invaluable in diagnosis, management, and prognosis. This technology has enormous potential in cancer diagnosis.

Similarly, Li and colleagues developed PAN fibres utilising 3-aminopropyltriethoxysilane as the electrospinning precursor. The presence of amine groups on the nanofiber surface facilitated negatively charged nanoparticles to be conjugated. In this study, platinum was employed as a negatively charged loading molecule. Platinum, as a radiosensitiser, is a common chemotherapeutic agent utilised in HNC. It is also used in the photothermal treatment of malignancy. A high nanofiber loading rate (5.61%) was achieved secondary to evenly distributed, cationic amine groups [73].

Chen and colleagues successfully demonstrated the anti-tumour activity of PLA nanofibers loaded with 15% titanocene dichloride in vitro. Utilising lung SPAC-1 cancer cells, researchers demonstrated that titanocene and loaded titanocene (PLA nanofibers + titanocene) inhibited cancer cell development [74]. Additionally, Ignatova and colleagues produced electrospun poly(L-lactide-co-D, L-lactide) fibres loaded with quaternised chitosan together with DOX on HeLa cells, discovering that these fibres attenuate cellular activity with greater potency than DOX alone for the first 6 h [75]. This research identified that these electrospun fibres, loaded with quaternised chitosan, were effective when utilised in conjunction with DOX to treat human breast cancer cell lines. In these experiments, drug-loaded quaternised chitosan significantly inhibited the proliferation of carcinoma cells, suggesting electrospun scaffolds may be used successfully for at least some cancers [76].

Luo and colleagues examined the anticancer effect of electrospun PEG-PLA nanofibers containing hydroxycamptothecin (HCPT) with mouse hepatoma H22 cells and found that HCPT was initially released in bursts followed by a period of sustained release in vitro [77]. Results showed that HCOT was highly sensitive secondary to a lactone ring, with up to 90% of the loaded drug released after 20 days’ incubation, and PEG-PLA fibre degradation increased in the presence of the drug. This loaded nanofiber composite subsequently demonstrated effective cytotoxicity towards H22 cancer cells in an in vivo murine model.

Deng and colleagues produced nanofibers by electrospinning thermoresponsive pNIPAM in a core–sheath arrangement with pNIPAM as the shell and polylactic acid PLA as the core [78]. PLA nanofibers were prepared in the presence of combretastatin A4 (CA4), a tubulin polymerisation inhibitor, and N,N’-methylenebisacrylamide cross-linker. The study found that decreasing the critical solution temperature varied drug release. When below the lower critical solution temperature (LCST), the rate of CA4 release was mitigated by the pNIPAM shell, with the rate of drug release increasing significantly when greater than the LCST.

Researchers have recently identified techniques to stimulate electrospun nanofibers to respond to different tumour microenvironment conditions to become active. Acidity, reactive oxygen species, light and magnetic fields have all been shown to enable targeted activation of scaffolds and mediate tumour cell death [79,80,81].

## 13. Chelation of Immunotherapies to Electrospun Nanofiber Scaffolds

Compared with traditional HNC therapeutic strategies, namely surgery, radiotherapy and platinum-based chemotherapy, new immunotherapeutic agents, specifically humanised antibodies targeting the PD-1-PD-L1 cell receptor system, have heralded a paradigm shift in treatment. Known as immune checkpoint inhibitors (ICI), these have demonstrated improved efficacy and lower toxicity in patients with advanced HNSCC that is recurrent or metastatic [82,83,84,85]. PD-1 is targeted by nivolumab and pembrolizumab, whilst avelumab, atezolizumab, and durvalumab are approved PD-L1 inhibitors [86,87].

PD-1 is mainly expressed in T cells, and the PD-1 pathway plays a role in regulating previously activated T cells [88]. PD-L1 and PD-L2 are expressed by a variety of tumours, including HNSCC [89]. The interaction of PD-L1/PD-L2 with PD-1 on T cells leads to immune evasion [88]. Furthermore, elevated levels of PD-1 are biomarkers for T cell exhaustion, a differentiation state in chronically stimulated T cells; this state is linked with the loss of T cell function [90]. However, resistance is currently a major limitation of targeting PD-1. Mechanisms of resistance are complex and include expression of multiple immune checkpoints that suppress T cell function (such as LAG-3 and TIM-3), deficiency in antigen presenting machinery (APM), and gene expression (such as PI3K/AKT and JAK2 mutations) leading to T cell exhaustion [91]. Indeed, pembrolizumab has a response rate of only 15% in HNSCC [92].

To date, although antibodies have recently been chelated to electrospun nanofibers scaffolds for the detection of illicit medications [93], and their combination has also been raised as a potential benefit for biosensing analytical tools [94], no literature currently exists for their potential as a combination therapy in HNC. Given the electrospinning environment, post-treatment chelation of antibodies to the nanofiber scaffold may be most appropriate; however, fibre specificity for the Fc domain of the antibody would be required. Certainly, targeted delivery to the tumour and/or tumour bed may help attenuate adverse events observed with systemic delivery of antibody immunotherapies.

Alternatively, small molecule immunotherapies (SMIs) may offer characteristics more amenable to nanofiber chelation and sustained release profiles. SMIs are potentially less expensive than antibodies and can target intracellular signalling and transcriptional pathways and mechanisms upstream of PD-1-PD-L1 receptors expressed on the cell surface. Table 1 provides details in respect to clinical trials that have been conducted using SMIs for HNC. These strategies have targeted a range of key mediators, spanning transcription factors (signal transducer and activator of transcription 3, aryl hydrocarbon receptor, peroxisome proliferator-activated receptor-alpha) and stimulator of interferon genes (STING), an adaptor protein that induces the secretion of type I interferons and proinflammatory cytokines. Delivering these directly to the tumour and/or tumour bed will increase the concentration of SMI delivered to the tissue and may also overcome issues with hydrophobicity associated with oral delivery techniques, metabolism, and tissue distribution.

Neutrophil extracellular traps (NETs) have recently been demonstrated to play an integral role in cancer metastasis [95]. Multiple NET-based therapies are currently being tested in pre-clinical and clinical models and may also provide an opportunity for chelation to electrospun nanofiber scaffolds for delivery to the post-resection tumour bed to mitigate the potential for tumour metastasis. Table 2 provides a summary of agents targeting NETs in different phases of development.

## 14. SMI Nanofiber Scaffold Delivery to the Difficult-to-Access Primary HNC

The development of bioadherent, sustained release nanofibers may provide an opportunity for anti-tumour therapies, specifically SMIs, that can be delivered by minimally invasive techniques to the primary tumour to promote immunorecognition and tumour lysis. This may overcome the necessity for functionally and aesthetically destructive surgical or radiotherapy techniques targeting areas that are difficult to access, namely the sinonasal vault, nasopharynx, and larynx.

Targeted delivery to the tumour site would avoid excessive systemic drug circulation, attenuating off-target side effects to the tissues of the body. Sustained compound release enables therapeutic dose delivery to the primary site whilst limiting systemic drug concentration. Local release kinetics will need to be determined for a given drug. An understanding of biological interactions with local tissue would also help in avoiding systemic side effects Additionally, this may reduce the necessity for repeated drug administration, commonly used in current systemic chemotherapy or immunotherapy regimes [96].

Immunotherapy chelated nanofiber scaffolds could be delivered endoscopically to early-stage (T_1_ or T_2_) tumours of these sites, releasing the brakes of the immune system within the tumour microenvironment [97]. Endoscopic delivery (nasendoscopy or microlaryngoscopy) could involve a direct application to the affected tissue bed (as an ‘on lay’ to the tumour) or after resection of the tumour (i.e., laser resection of early laryngeal cancer or ‘piecemeal’ resection of a sinonasal or anterior skull base tumour) to the resection bed. Given recently identified neoadjuvant benefits of immunotherapy in melanoma, oral cavity SCC (OCSCC) and locoregionally advanced OCSCC, oropharynx, hypopharynx and larynx [98,99,100,101,102], there may even be potential to utilise this technique to facilitate tumour shrinkage prior to surgical intervention or even generation of tumour targeting memory cells to mitigate future recurrence and improve disease-free and overall survival [103,104]. There may also be potential for a similar device to be developed for delivery to cutaneous malignancies.

This approach may also mitigate the risk of adverse events caused by systemic delivery of immunotherapy agents. Adverse events have been reported in 68.2% of patients receiving monoclonal antibody immunotherapies, with grade III/IV events occurring in 10% of recipients [105,106], even after 2 years [107].

## 15. Nanofiber Scaffold Delivery to Support Immunorecognition in the Positive Margin

Ranging from 10.8 to 22.7% of cases, positive post-operative surgical resection margins remain a significant issue in the management of HNC [104,108,109]. Positive margins are associated with a worse prognosis, a local recurrence rate of 90%, and a reduction in 5-year overall survival to 10% in oral cavity cancer [110,111,112,113,114]. A recent study has demonstrated that radiotherapy does not improve survival in positive margin cases [115]. Higher positive margin rates in HNC likely represent the balance between providing a clear surgical margin and reducing the necessity to remove functionally important structures, compared to surgical ablation of cancers in other areas of the body.

Although likely to be multifactorial, these poor outcomes may, at least in part, be attributable to the repopulation phenomenon, dormant tumour cell activation and exacerbation of tumour cell migration caused by NET-mediated inflammatory cell processes associated with wound healing and loss of tissue boundaries secondary to surgery [116,117,118,119,120,121,122]. Certainly, elevated concentration of circulating NETs in the post-operative period has been associated with an increased risk of recurrence and metastasis [123,124,125]. High-risk positive margins are an appropriate group in which to evaluate the potential utility of nanofiber immunotherapy chelated scaffolds to the primary and regional surgical beds to aid immunorecognition of the tumour whilst also reducing tumour cell metastasis.

Localised delivery of small molecule immunotherapy scaffolds could provide new opportunities for synergistic or adjuvant therapy in current treatment modalities and help to control residual locoregional cancer prior to delivery of radiotherapy or chemoradiotherapy. There may also be potential for this approach to provide long-term cell-mediated immunological memory, reducing the risk of tumour recurrence. Scaffold degradation duration could be associated with adverse delivery of adjuvant radiotherapy. Whether a degradable scaffold may affect tissue penetration or cause beam scatter would be an added important consideration in determining the feasibility of incorporating scaffold delivery into treatment strategies.

## 16. Limitations

Like all new therapeutic strategies, it is difficult to determine all the potential issues that may arise; however, several potential challenges can be extrapolated from previous interventions associated with the insertion of biomaterials into human tissue beds.

### 16.1. Foreign Body Reaction

Implantation of foreign material often leads to an inflammatory and fibrotic process mediated by an immune system attempting to degrade it [126]. Although it is hoped that a nanofiber scaffold degrades within a short period, limiting regional fibrosis, it remains a possibility. Regions of ongoing inflammation and or fibrosis may affect the interpretation of diagnostic imaging tests (i.e., FDG PET/CT), making it difficult to exclude persistent and or recurrent disease [127]. Animal studies to investigate degradation rate, and locoregional immunological and fibrotic reactions will help to quantify this issue.

### 16.2. Adjuvant Therapy

Scaffold degradation duration could also be associated with adverse delivery of adjuvant radiotherapy. Whether a degradable scaffold may affect tissue penetration or cause beam scatter resulting in under- or overdosing of ionising radiation contributing to treatment failure would also be an important consideration in determining the feasibility of incorporating scaffold delivery into radiotherapy-associated treatment strategies. Certainly, this has been a previous issue with titanium in the reconstruction of the craniofacial skeleton and materials like glass fibre-reinforced composite and polyether ether ketone to a lesser degree [128]. Preclinical radiochromic and diamond detector assessment would aid in identifying whether scaffolds affected radiation delivery characteristics.

### 16.3. Immunotherapeutic Sustained Release Profile and Locoregional Tissue Perfusion

Optimising release kinetics to ensure effective dosing and tissue penetration will also require thorough assessment. Head and Neck subsite delivery will also need to be considered, as release dynamics within the sinonasal vault may differ significantly from the soft tissue compartment of the neck. Further to this, the effect of vasculogenesis and regional tissue perfusion will also affect diffusion characteristics and locoregional tissue concentrations of a given immunotherapeutic. The high surface area of scaffolds improves compound loading, while nanofiber length, chemical composition, diameter, and surface functionalisation have been shown to influence drug absorption rates, which can be optimised for specific disease applications and tissue types [129,130,131].

### 16.4. Endoscopic Delivery

Characteristics associated with easy delivery will also need to be explored. Will specific instruments be required, will it emulsify, disintegrate, or fragment upon contact with tissue or fluids, or will it be difficult to distribute over the tissue bed and all considerations that will require assessment and optimisation.

Consequently, preclinical studies will play a significant role in determining efficacy and helping to understand and overcome some of these potential challenges.

## 17. Personalising SMI Electrospun Scaffolds

### 17.1. Clinical Scenarios

Scaffolds could be beneficial as a personalised therapeutic option in several patient groups, namely patients unsuitable for standard-of-care therapy (i.e., patients with multiple medical co-morbidities and advanced age) or patients with tumours not amenable to surgical intervention (i.e., locally invasive or near important functional structures). SMI Scaffolds could also be beneficial in tumours deemed too large for resection, where a reduction in size after therapy may enable subsequent surgical intervention.

### 17.2. Specific Biomarkers

Biomarkers are gaining importance in aiding clinical decision-making and personalising therapeutic strategies. Although there is a wide variety of tumour biomarkers currently being investigated within the HNC space [132], at this time, tumour PD-L1 expression and tumour mutation burden (TMB) are being used clinically to help guide immunotherapeutic decision making [133,134]. The American Society for Clinical Oncology (ASCO) recently published a guideline supporting the benefit of quantifying these biomarkers in tumour specimens [135]. Although there has been a focus in this article towards SCC associated HNC, there is also potential to utilise nanofiber scaffolds in differentiated thyroid cancer. Tyrosine receptor kinase inhibitors targeting RET and BRAF pathways (i.e., sorafenib and lenvatinib) could also be chelated to scaffolds and delivered to the surgical bed to mitigate systemic side effects [136].

**Table 1:** SMIs in HNC clinical trials [137].

Drug	Target	Phase	n	NCT ID	Objective	Results
BAY2416964	AHR	1	78	NCT04069026 [138]	Safety/tumour response study in advanced HNSCC	Safe and demonstrated promising anti-tumour activity in previously treated patients
TPST-1120	PPAR-a	1/1b	38	NCT03829436 [139]	TPST-1120 in combination with Nivolumab vs. TPST-1120 alone for advanced HNSCC	Combination therapy superior to monotherapy with predominately acceptable adverse event profile
AZD9150	STAT3	1b/2	30	NCT02499328 [140]	ASD9150 with Duravalumab vs. Duravalumab alone; in recurrent or metastatic HNSCC refractory to platinum-based chemotherapy	Combination therapy superior to Duravalumab alone with an acceptable adverse event profile
MK-1454	STING	1	157	NCT03010176 [141]	MK-1454 (ulveostinag) with concurrent pembrolizumab vs. MK-1454 alone for advanced stage HNSCC	Concurrent therapy superior to monotherapy alone with an acceptable adverse event profile

**Table 2:** Potential NET-Based Therapies.

Target	Drug	Intended Mechanism of Action	Potential Impact on Tumour Metastasis
Histones	STC3141 [142], Unfractionated Heparin [143]	Small/large polyanions that interact electrostatically with histones, thereby neutralising their pathological effects	Inhibition of histone-dependent pro-tumorigenic pathways such as TLR4/histone-dependent immunosuppression in TME, histone-dependent endothelial or platelet activation/thrombosis that help confer tumour survival and metastatic ability
Neutrophil Elastase (NE)	Sivelestat [144]	Competitive inhibitor of the NET-expressed serine protease	NE is integral to NETosis and has been shown to attenuate hepatic metastasis in a preclinical model of colorectal cancer
CXCR1/2:IL-8	SX-682/Avarixin [145,146], NCT03161431, NCT03473925	Neutrophil chemotaxis and NETosis inhibition	Suppressing myeloid cell recruitment
PKC	Metformin [147,148,149,150,151]	Attenuates NETosis by inhibiting PKC	Studies show metformin-mediated reduction in circulating NET markers and abrogation of NET promoted carcinogenesis in preclinical models
COX-1	Aspirin [152,153,154]	Inhibition of platelet-dependent expression of neutrophil chemokines CXCL4 and CCL5	Studies have demonstrated the anti-metastatic effects of ameliorating NET production via COX-1 inhibition.
DNA	Dornase alfa [155,156]	Cleaves extracellular DNA	rhDNase 1 has been shown to attenuate metastasis in preclinical models of lung, breast, and pancreatic cancer

## 18. Concluding Remarks

Overall survival for advanced HNC has improved little over the past 30 years; the financial burden for health systems is high, and intervention commonly exacerbates tissue destruction. Antibody-based immunotherapies have provided a new therapeutic approach for this unmet clinical need. However, response rates in HNC patients are low and resistance to therapy can develop. The emerging field of SMIs may represent a complementary or even an alternative approach. Recent developments in nanomaterial technology, notably the growing use of electrospun nanofiber scaffolds in conjunction with SMIs and other anti-tumour agents, are poised to provide a paradigm shift for targeted therapeutic strategies in HNC, overcoming issues associated with systemic adverse reactions, cost, and drug concentrations.

## Data Availability

Not applicable.
